# Haralick texture feature analysis for quantifying radiation response heterogeneity in murine models observed using Raman spectroscopic mapping

**DOI:** 10.1371/journal.pone.0212225

**Published:** 2019-02-15

**Authors:** Irene Vrbik, Samantha J. Van Nest, Phiranuphon Meksiarun, Jason Loeppky, Alexandre Brolo, Julian J. Lum, Andrew Jirasek

**Affiliations:** 1 The Department of Statistics, University of British Columbia Okanagan Campus, Kelowna, BC, Canada; 2 The Department of Physics and Astronomy, University of Victoria, Victoria, BC, Canada; 3 The Department of Physics, University of British Columbia Okanagan Campus, Kelowna, BC, Canada; 4 The Department of Chemistry, University of Victoria, Victoria, BC, Canada; 5 Trev and Joyce Deeley Research Centre, BC Cancer, Victoria, BC, Canada; 6 Department of Biochemistry and Microbiology, University of Victoria, Victoria, BC, Canada; Beijing University of Technology, CHINA

## Abstract

Tumour heterogeneity plays a large role in the response of tumour tissues to radiation therapy. Inherent biological, physical, and even dose deposition heterogeneity all play a role in the resultant observed response. We here implement the use of Haralick textural analysis to quantify the observed glycogen production response, as observed via Raman spectroscopic mapping, of tumours irradiated within a murine model. While an array of over 20 Haralick features have been proposed, we here concentrate on five of the most prominent features: homogeneity, local homogeneity, contrast, entropy, and correlation. We show that these Haralick features can be used to quantify the inherent heterogeneity of the Raman spectroscopic maps of tumour response to radiation. Furthermore, our results indicate that Haralick-calculated textural features show a statistically significant dose dependent variation in response heterogeneity, specifically, in glycogen production in tumours irradiated with clinically relevant doses of ionizing radiation. These results indicate that Haralick textural analysis provides a quantitative methodology for understanding the response of murine tumours to radiation therapy. Future work in this area can, for example, utilize the Haralick textural features for understanding the heterogeneity of radiation response as measured by biopsied patient tumour samples, which remains the standard of patient tumour investigation.

## Introduction

Radiation therapy is a standard treatment for approximately 50% of all cancer patients [1]. While significant improvements in the technological development of radiation therapy have occurred in the past several decades, a number of challenges in treatment efficacy remain unmet. Among these challenges, optimizing, or personalizing, radiation therapy remains difficult due to the considerable inter- and intra-patient heterogeneity of response to radiation [2]. Indeed, heterogeneity of radiation response can exist within individual tumours, and can lead to differential patient response [3–5].

However, the precise mechanisms in which tumours establish radioresistance depend on numerous factors. For example, in radiation biology, it is well established that oxygen can provide the cell with a source of reactive species to generate DNA-damaging radicals. Moreover, oxygen may also contribute to the fixation of DNA damage once the initial insult has been established. It is fair to say that the complete mechanisms of radiation resistance and response in tumours is a complex combination of factors, and although differential responses to radiation therapy have been observed in the clinic for decades, the molecular basis of such responses remains an enigma. For a variety of cancers, recent studies have unequivocally highlighted the significant molecular heterogeneity that exists in patients’ tumours and in tumour radiation response [6].

Tumour heterogeneity remains a challenge to measure in any scenario. Although a number of assays have been proposed in the literature, no one technique has proven to provide a comprehensive and clinically viable assessment strategy [7]. In previous investigations, we have demonstrated that Raman spectroscopy can offer multiplexed, biologically significant molecular-level information on cellular and tumour radiation response [8]. We have demonstrated that Raman mapping can be used to, for example, provide spatially resolved information on glycogen production in murine models of H460 lung tumours post irradiation [9]. However, the quantification of tumour response heterogeneity is challenging owing to architectural complexity, temporal changes, spatial variation, inherent subpopulations within host that are part of the tumour environment, and potential inaccuracies in data collection, just to name a few.

To overcome the issue of heterogeneity, it has been suggested that the average spectra be used as a representative of the target population, [1, 2]. However, such strategies by their very nature lose information on the spatial origin of given biomolecular components, and is true for genomic studies in cancer. Textural analysis attempts to quantitatively describe characteristics of images based on the spatial arrangement of intensity values. While it has been established in pattern recognition [10], and image processing [11], it more recently has been finding application in the biomedical field [12–16]. For instance, textural features of PET scans extracted pre- and post-treatment from patients with esophageal cancer have been used to differentiate between nonresponders, partial responders, and complete responders [15]. Moreover, the use of PET imaging relies on tumor uptake of the radiotracing compound which could be impacted by the profusion of the microenvironment. In other work, textural measurements (such as heterogeneity, contrast, and energy) were observed to correlated with the fracture toughness of bone tissue [16]. While there is great potential for image analysis to improve our understanding of complex systems like tumours [4, 5], this remain an active area of research.

Using the techniques described by Haralick [17], this paper calculates several textural features on Raman spectral maps. Besides using them to gauge tumour heterogeneity, these quantities are employed to assess dose response in murine models of lung tumours irradiated with clinically relevant doses of ionizing radiation. The next section outlines the Raman spectroscopy data, as well as the details on how the spectral maps were generated. Subsequent sections provide the specific definitions for select Haralick features and explore whether certain characteristics of these tumours change in response to radiation dose and time post irradiation.

## Materials and methods

**Fig 1:**
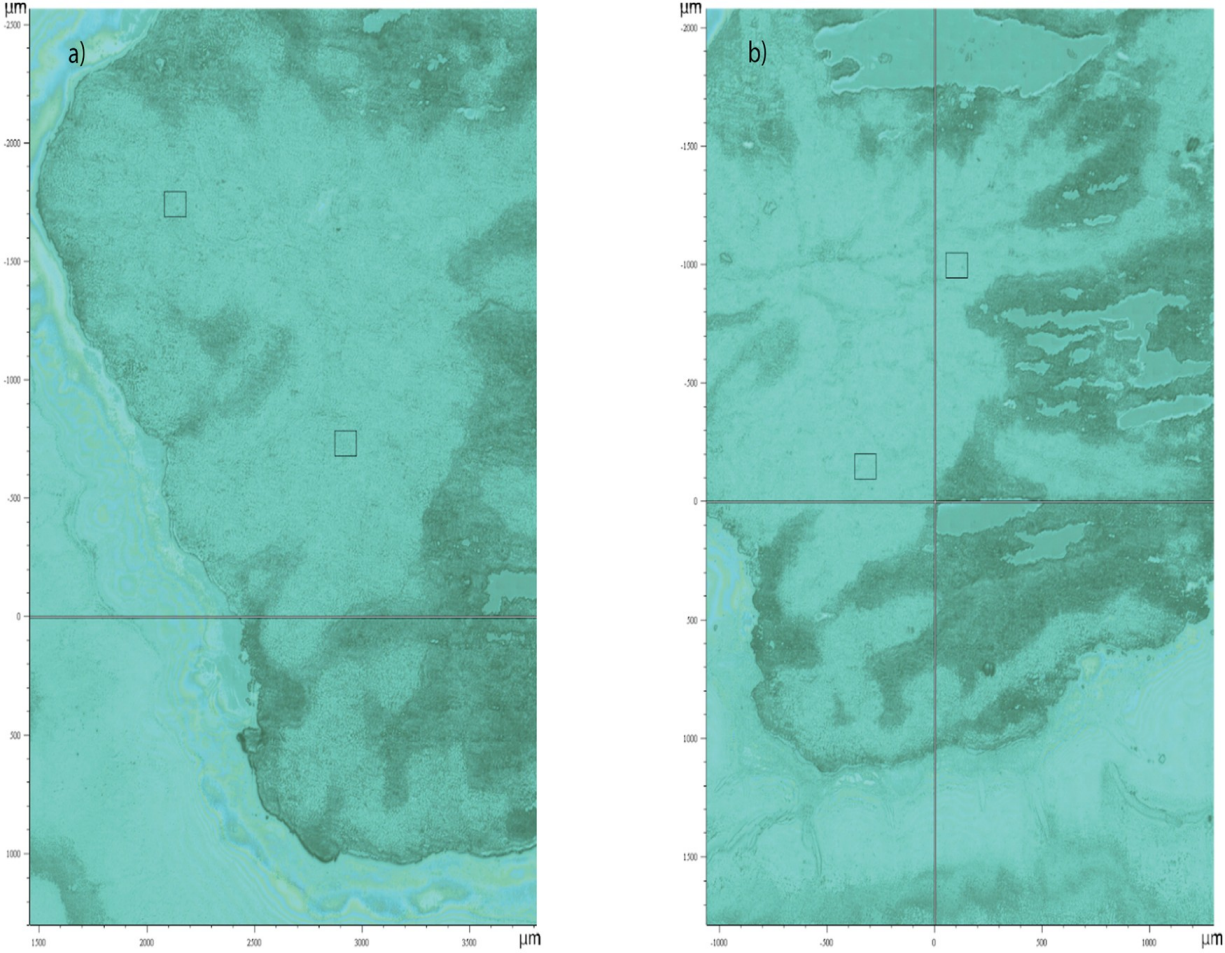
White light images of a H460 tumour xenograft, showing two sections from the same tumour. Mapped tissue regions are outlined by black squares, showing the 100 um x 100 um area analyzed by Raman spectroscopy. Mapped regions were selected at random.

All mouse protocols, tumour growth parameters, irradiations, tumour sectioning, and Raman analysis were performed as described previously [9]. Briefly, H460 tumours were implanted and grown in murine models. Four mice were irradiated to 5 Gy, four mice were irradiated to 15 Gy, and a sham group (four mice) were left as controls, for a total of 12 mice. Tumours were harvested 3 days post irradiation and sectioned into slices using established protocols [9]. In terms of Raman analysis of resulting tumour sections, each Raman spectrum was collected utilizing a Renishaw Raman microscope (Renishaw Inc, Chicago, USA) operating at 785 nm laser excitation, a 100x dry objective, and a 10s acquisition time. Raman maps were collected on up to 4 separate tumour slices for each of the 12 mice in the study and were acquired on a 15 × 15 *μ*m grid. The slices were selected randomly, as described in our previous work [9] and shown by way of illustrative example in Fig 1. In all cases 100 × 100 or 200 × 200 micron regions were studied.

Post-Raman acquisition spectral processing included cosmic ray removal, spectral smoothing, baseline subtraction, and volume normalization as described previously [8]. The only minor deviation here is within the baseline correction algorithm where we here use the baseline package for spectra data available in R [18] using the second derivative constrained weighted regression [19] (method = “als”) with the second derivative constraint, lambda, equal to 4. Our complete data set, **X**_*n*×*p*_, contain *n* = 6581 spectra comprised of 74 Raman maps with a grid size of either 8 × 8 (15 *μ*m × 15 *μ*m per pixel) or 14 × 14 pixels (20 *μ*m × 20 *μ*m per pixel). That is, each map is stored in either 64 or 196 rows in **X**_*n*×*p*_, where the row names of **X**_*n*×*p*_ contain the locational information for each spectrum. An example white light optical map illustrating regions of interest for Raman mapping is shown in Fig 1.

Principal Component Analysis (PCA) [20–22] is a commonly used technique for data reduction that allows the user to project a high-dimensional data set into a new set of variables in a lower dimensional space. PCA is an orthogonal transformation which ensures that each variable, henceforth referred to as principal component, is uncorrelated and ordered such that the first principal component explains as much of the variability in the data as possible, the second component has the second largest possible variance, and so on. Applying PCA to our data results in a score matrix **Y**_*n*×*q*_ with *q* ≪ *p* where each row represents the corresponding Raman spectrum transformed into the new PC coordinate system. Herein, we concentrate on the first principle component which explains approximately 55 percent of the variability in our data. In previous work we have established that PC component 1 in the irradiated H460 tumours used in the present study corresponds to glycogen production post irradiation [8] (see S1 Fig for a visual comparison of principal component 1 with the glycogen spectrum).

**Fig 2:**
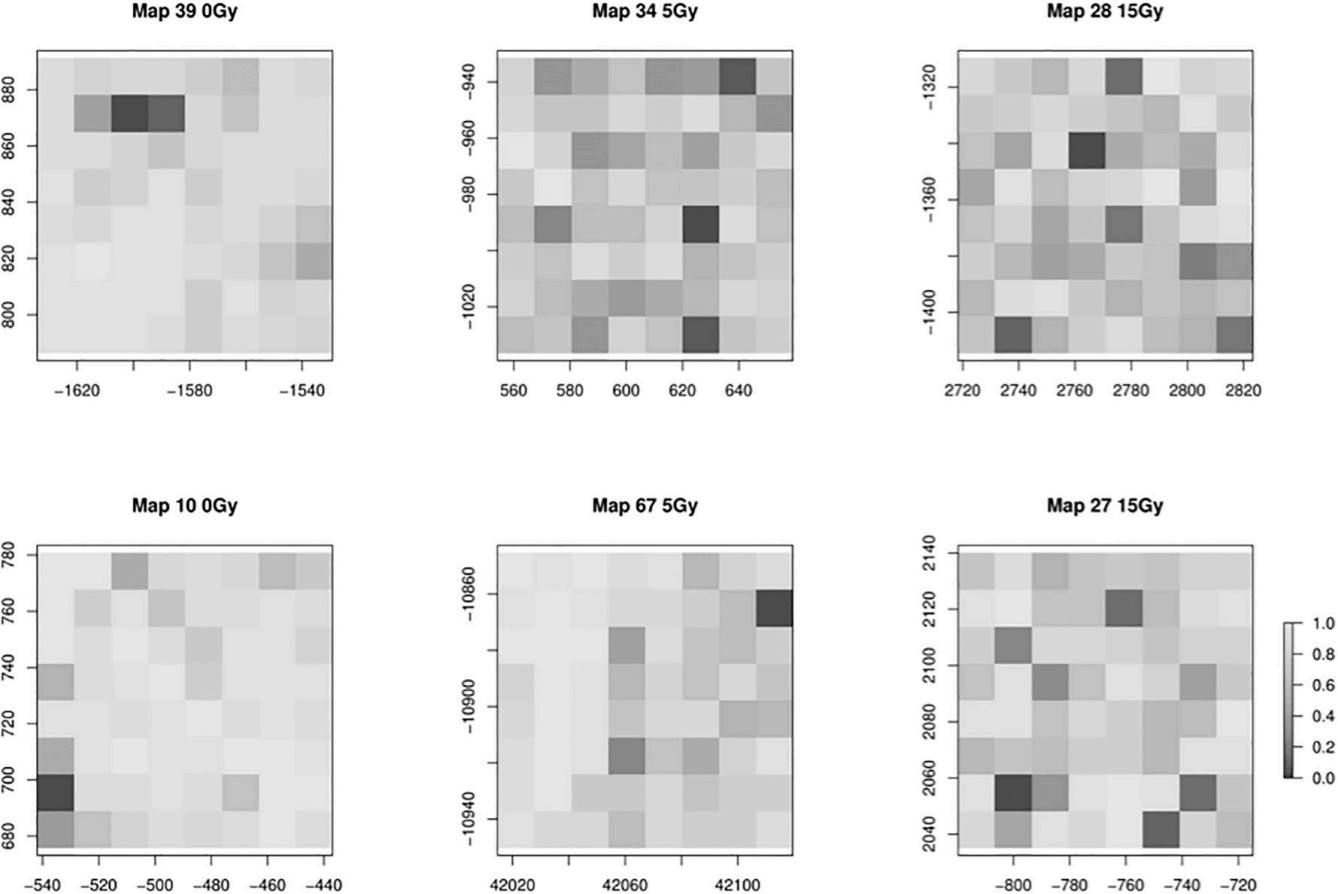
Example of six gray-scale Raman maps with pixel intensities equal to the scaled and discretized PC1 scores (glycogen production) obtained from running PCA on X_*n*×*p*_. Left: Map from non-radiated tumour section. Maps from irradiated mouse tumour sections are shown for mice irradiated to 5 Gy (middle) and 15 Gy (right). Pixels are 15 × 15 *μ*m. Map numbers refer to tumour slice region of interest. A representative sample of maps has been shown.

**Fig 3:**
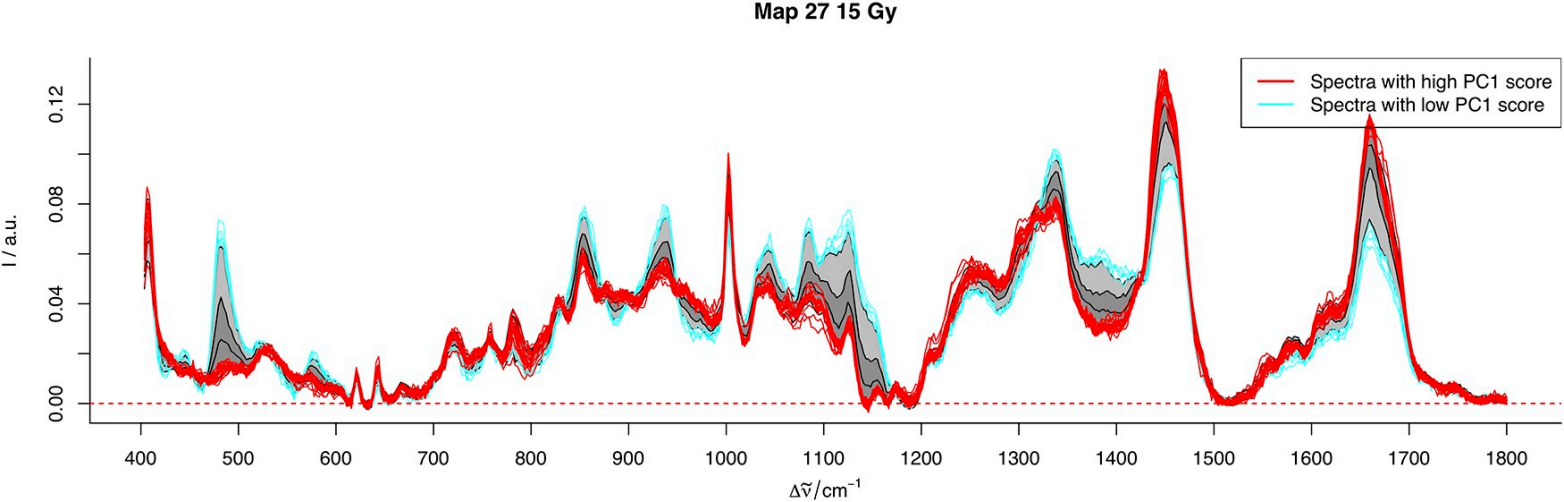
The Raman spectra from Map 27. The light gray bands indicate ± 1 standard deviation while the darker gray indicate the 5th and 95th percentile.) The spectra corresponding to low and high PC 1 score values are plotted in blue and red, respectively.

To generate our 2D Raman images (see Fig 2 for example and S2–S4 Figs in the supplementary data for the complete collection), we populate the grid matrices described above with PC component 1 scores (i.e. glycogen production scores) scaled between 0 and 1. In essence, the gray scale image provides a visual representation of tumour glycogen levels. As an example, Fig 3 represents how the pixel values for Map 27 relates to the Raman spectra. Spectra having high and low glycogen production scores are plotting in red and blue, respectively. We remark that the spectral interpretation in certain areas having a large discrepancy between red and blue spectra (eg. 490, 850, 1050 cm^−1^) correspond to spectral peaks characterized by glycogen [23].

The original paper by Haralick [17] introduced a total of 14 measurements; however, many of these values are highly correlated rendering all but five useful in practice [24, 25]. These features rely on a gray level cooccurrence matrix (GLCM), denoted here by **M**, which contains the textural information of an image. Assuming we have a gray-scale image composed of *N*_*g*_ gray levels, the *ij*th element of GLCM, denoted *M*(*i*, *j*), counts the number of times a pixel with the value of *i* is neighbouring a pixel with the value of *j*. Neighbours are defined by a user-specified distance *d* and angle *θ*. Finally, the GLCM is normalized so that the total sum of the elements sum to 1. We shall denote the elements of this *N*_*g*_ × *N*_*g*_ normalized GLCM by *m*_*d*,*θ*_(*i*, *j*), where we can write *m*_*d*,*θ*_(*i*, *j*) as (for the example case of *θ* = 0°) [17]:

$$\begin{array}{c} m_{d,0^{{}^{\circ}}}\left(i,j\right)=\#\{\left(\left(k,l\right),\left(m,n\right)\right)\;\;\epsilon \left(N_{y}\times N_{x}\right)\;\times \;\left(N_{y}\times N_{x}\right)\\ |k-m|=0,\;\;|l-n|=d\\ I(k,l)=i,\;\;I(m,n)=j\} \end{array}$$ (1)

Following the recommendations in [25], we focus on *homogeneity* (H), *contrast* (Con), *correlation* (Cor), *entropy* (E) and *local homogeneity* (LH). These texture features are calculated as follows:

$$\begin{array}{ccc} \text{H} & = & \sum_{i=1}^{N_{g}}{\left(m_{d,\theta }\left(i,j\right)\right)}^{2}, \end{array}$$ (2)

$$\begin{array}{ccc} \text{Con} & = & \sum_{i=0}^{N_{g}-1}k^{2}\{\sum_{i=1}^{N_{g}}\sum_{j=1}^{N_{g}}\delta_{\left|i-j\right|}m_{d,\theta }\left(i,j\right)\}, \end{array}$$ (3)

$$\begin{array}{ccc} \text{Cor} & = & \frac{\sum_{i=1}^{N_{g}}\sum_{j=1}^{N_{g}}\left(ij\right)m_{d,\theta }\left(i,j\right)-\mu_{x}\mu_{y}}{\sigma_{x}\sigma_{y}}, \end{array}$$ (4)

$$\begin{array}{ccc} \text{E} & = & -\sum_{i=1}^{N_{g}}\sum_{j=1}^{N_{g}}m_{d,\theta }\left(i,j\right)\log\left(m_{d,\theta }\left(i,j\right)\right), \end{array}$$ (5)

$$\begin{array}{ccc} \text{LH} & = & \sum_{i=1}^{N_{g}}\sum_{j=1}^{N_{g}}\frac{1}{1+{\left(i-j\right)}^{2}}m_{d,\theta }\left(i,j\right). \end{array}$$ (6)

where *N*_*g*_ is the number of distinct pixels in the Raman map, and $\mu_{x}=\sum_{i=1}^{N_{g}}\sum_{j=1}^{N_{g}}i\cdot m_{d,\theta }\left(i,j\right)$, $\mu_{y}=\sum_{i=1}^{N_{g}}\sum_{j=1}^{N_{g}}j\cdot m_{d,\theta }\left(i,j\right)$ and $\sigma_{x}=\sqrt{\sum_{i=1}^{N_{g}}\sum_{j=1}^{N_{g}}{\left(i-\mu_{x}\right)}^{2}m_{d,\theta }\left(i,j\right)}$, $\sigma_{y}=\sqrt{\sum_{i=1}^{N_{g}}\sum_{j=1}^{N_{g}}{\left(j-\mu_{y}\right)}^{2}m_{d,\theta }\left(i,j\right)}$. To calculate the GLCM and Haralick features we used the glcm() and haralick functions available in the R package wvtool [26].

Kumar and Sreekuma, 2014 [27] provide some helpful comments on how interpret the Haralick texture features. Briefly, homogeneity will be close to 1 when only a few dominant gray-tones are present. Contrast, will be equal to 0 for a constant image and become larger as the pixel intensities in a local neighbourhood become more disparate. Correlation ranges from -1 to 1 and reflects how correlated a pixel is to its neighbours. Entropy reflects the amount of randomness in intensity of an image and will increase with the images local complexity. Local homogeneity measures the similarity of pixels and is larger when there are minimal local changes.

## Results

For each of the 2D gray-scale Raman images, the Haralick textural features given in Eqs (2)–(6) were calculated. Since the correlation between pixels is likely to decrease with distance, we use *q* = 1 in accordance with [28]. To avoid any directional dependency, we averaged the values across four angles *θ* = 0, 45, 90, and 135° [29].

For illustration, we have provided the Haralick features corresponding to the maps displayed in Fig 2. Images that appear more monochromatic (eg. Map 10) have higher values of H and LH than images having more pixel variation (eg. Map 34). Although Map 10 and 39 are similar in terms of homogeneity, the neighbouring pixels in image 10 are closer in gray-tone and consequently yield a lower value for contrast. Map 39 and 67 have a higher measure of gray-tone linear dependencies and consequently produce larger correlation values. On the contrary, the lack of any discernible linear pattern in Map 28, for example, generates a correlation closer to zero. Finally, images with larger entropy values exhibit more randomness in pixel intensity. Representative results are summarized in Table 1.

**Table 1:** Summary of textural results for representative Raman maps of glycogen production in tumour section pre and post irradiation. H = homogeneity, Con = contrast, Cor = correlation, E = entropy, LH = local homogeneity.

Map	Dose	H	Con	Cor	E	LH
39	0Gy	0.0413	38.0764	0.2254	1.6355	0.4194
10	0Gy	0.0612	14.0903	0.0497	1.4351	0.4505
67	5Gy	0.0199	33.3194	0.1888	1.9419	0.2985
34	5Gy	0.0085	66.8472	-0.1985	2.1559	0.1193
28	15Gy	0.0059	77.1736	-0.1760	2.2696	0.1188
27	15Gy	0.0103	75.7153	-0.0498	2.0736	0.1718

**Fig 4:**
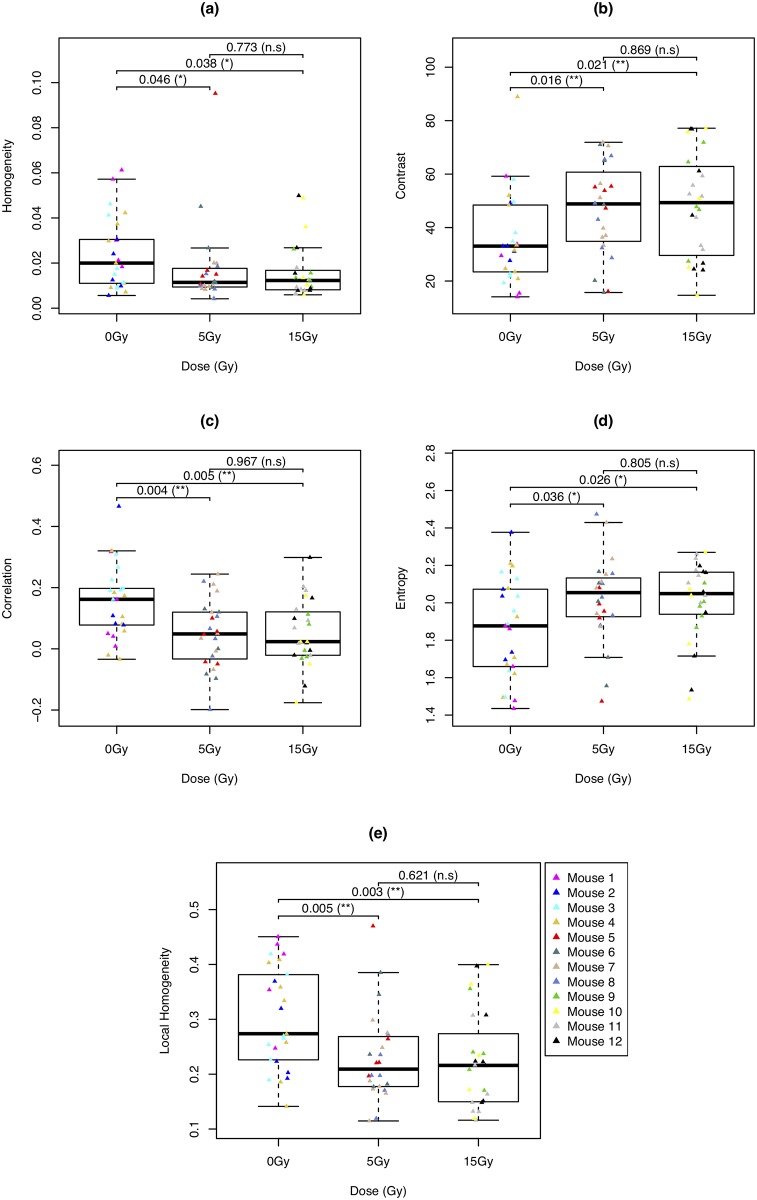
Side-by-side box plots for Haralick features. The box plots of the five Haralick features values obtained for the 74 gray-level Raman maps according to dose. The gray-level values are generated using the scaled PC1 scores. (a) homogeneity, (b) contrast, (c) correlation, (d) entropy, and (e) local homogeneity, as defined in Eqs 2–6. Dose levels are in gray units (Gy).

The Haralick features for the complete set of 74 gray-scale images are summarized in the box-and-whisker plots in Fig 4. Each of the twelve mice have 5—8 associated Raman maps; the legend provides a key to the mouse identification number. For each pair of box plots within each plot, a comparison for the difference of means is made using a Kruskal-Wallis test with a significance level of 0.05. Significant *p*-values are indicated using asterisks (* for values between 0.025—0.05 and ** for values between 0.001—0.025), while non-significant test are labeled as ‘n.s’. We shall denote $p_{i\_j}^{k}$ as the *p*-value for testing if the mean feature values obtained from Raman maps belonging to dose *i* is different than that of dose *j*, where *k*∈ {H, Con, Cor, E, LH} denotes the Haralick feature.

Among the 74 images considered, a significant difference between non-irradiated and irradiated groups was uncovered. For instance, the group of non-radiated mice has a significantly different mean contrast value than mice treated with 5 Gy and 15 Gy; $p_{0\_5}^{con}=0.016$, $p_{0\_15}^{con}=0.021$. Furthermore, there was no statistically significant difference between mice irritated with 5 Gy and 15 Gy; eg $p_{5\_15}^{con}=0.869$. This trend, which is prevalent among all Haralick features considered, indicates that the textural complexity of the images extracted from irradiated mice tends to be greater than those extracted from non-radiated mice. As these images were generated from the score values linked to glycogen, this work suggests that the affect of radiation on glycogen production is not uniform across the tumour.

## Discussion

The Haralick feature analysis provides a new methodology for shedding light onto the evolution of textural features within Raman maps of glycogen production within murine-irradiated tumours. For example, in this study we quantitiavely show a statistically significant decrease in the homogeneity of glycogen production (Fig 4a) as a function of irradiation dose. Put another way, glycogen production is heterogeneously distributed throughout the tumour and, furthermore, the extent of heterogeneity exhibits a radiation dose dependence. In a separate study we show that glycogen production is negatively correlated with tumour regression post radiation [30]. Furthermore, the current and past hypoxic state of local tumour morphology is correlated with glycogen production, thus affecting the spatial extent of tumour regression and glycogen production [30]. While we tackle the radiobiological implications of glycogen production in a separate work [30], it is clear that the Haralick features calculated here are (i) able to quantitate the extent of textural variation as a function of radiation dose, and (ii) correlate well with expected radiobiological trends in our murine models. Furthermore, it is apparent that glycogen heterogeneity exists not only intra tumour, but also between murine tumours. This variation is inherently interesting from the point of view of future assays based on tailoring treatment based on individual sample response and points towards future experiments dedicated to understanding this inter-murine variability.

**Fig 5:**
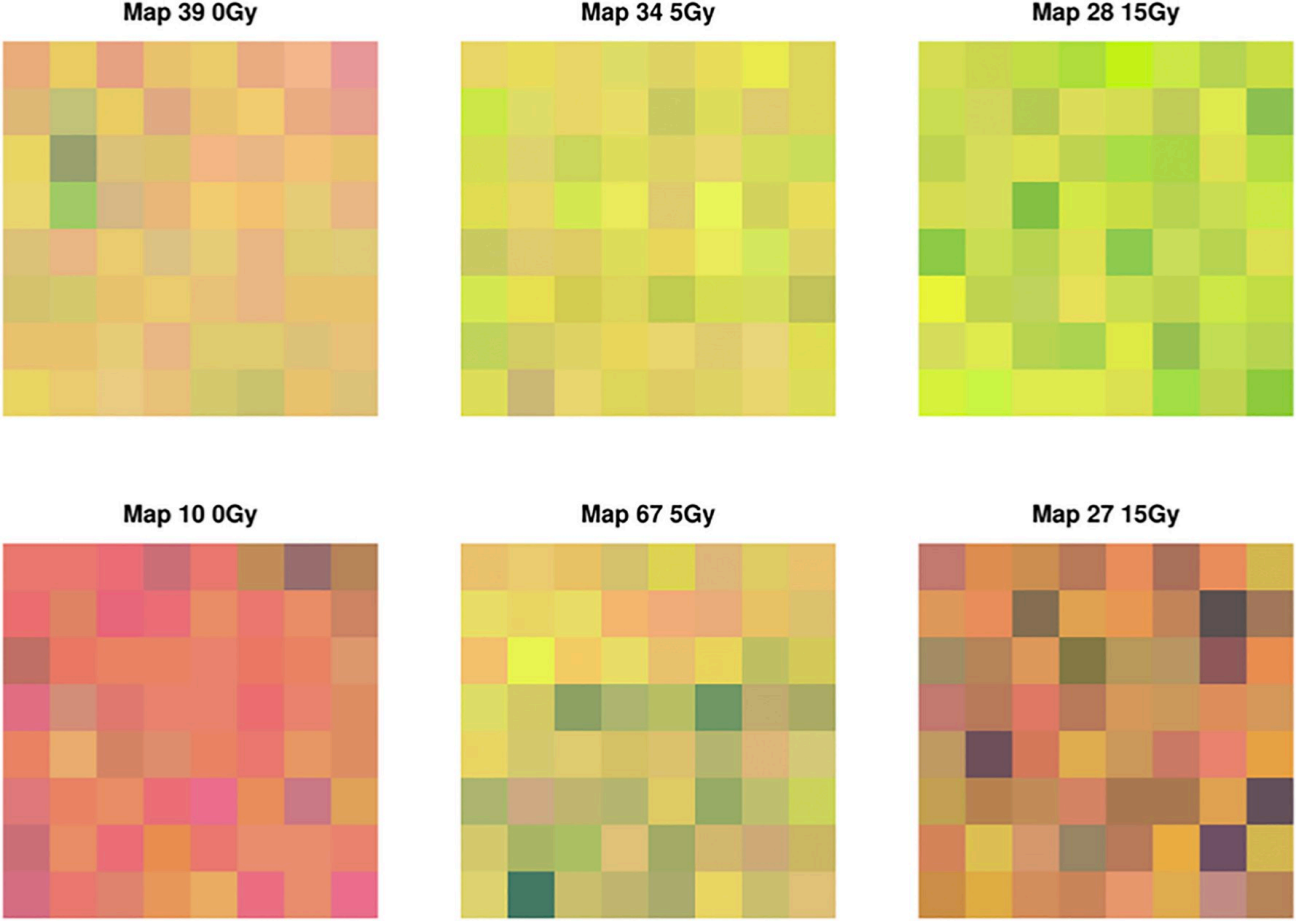
Example of six RBG-scaled Raman maps with red/green/blue intensities equal to the scaled PC1, PC2, and PC3 scores obtained from running PCA on X_*n*×*p*_. Left: Map from non-radiated tumour section. Maps from irradiated mouse tumour sections are shown for mice irradiated to 5 Gy (middle) and 15 Gy (right). Pixels are 15 × 15 *μ*m. Map numbers refer to tumour slice region of interest. A representative sample of maps has been shown.

Within our current approach, only a single principal component (PC) is considered. Although this component describes a large percentage (54.92%) of the total variability within the Raman dataset, the inclusion of additional PCs may be advantageous in terms of understanding variability of Haralick indices for PCs that may harbour additional biochemical information relating to radiation response. In order to analyze multiple PCs simultaneously, one could investigate an extension of Haralick textural features that uses the colours discriminators to describe colour images. In the context of this paper, rather than using a single PC score to create a gray-toned image, a pseudo-coloured Raman map could be generated using the first three PC scores to represent the red, green, and blue components in an RGB colour model; an example is provided in Fig 5. Owing to the fact that these principal components explain 79.34% of the total variability in our data, these images present a richer representation of the underlying spectroscopic information and could provide useful insight on the radiation-induced differences in succeeding PCs that may be linked to other biological interpretations. In future work, we will explore if and how the colour-indexed Haralick features are affected by this extension.

Haralick features provide possibilities for future implementation in the long term strategy of personalized radiotherapy. For example, it is possible that Haralick analysis on biopsy-based Raman maps can provide information on response heterogeneity that, along with orthogonal biological information, can be used in the assessment of overall response. Other in vivo image modalities provide information regarding tumor architecture as well as spatial context relative to normal adjacent or host tissue within an organ system. In some cases such as PET, indirect assessments of phenotype (e.g. glucose uptake) of a single biomarker can help infer some biological phenotype (e.g. metabolically active). While improvements may allow for potentially dual or even triple biomarker uptake analysis, these techniques are different than Raman spectroscopy. Raman spectroscopy provides a global picture of biochemical signatures across multiple biomolecules in a single sample and does not rely on uptake of a tracer. As such, the information collected from imaging and Raman spectroscopy are complementary in this regard rather than overlapping. Future Raman spectroscopic point-of-care probes under research development may in the future alleviate the need for biopsy samples. Further research is required to continue to develop this long term goal.

On a final note, we recognize that an array of texture analysis methods exist in the literature. We here do not presume that Haralick features will necessarily outperform other methods. Rather, we here demonstrate that textural features provide an excellent platform to tackle the issue of response heterogeneity. While this well-known model serves as a reasonable first choice in our exploratory analysis, alternative techniques such as the ones described in [31] could also prove useful. Additional work is required to establish the feasibility of other textural feature analysis techniques.

## Conclusion

The Haralick texture features display biologically relevant trends for dose response in tumours extracted from our murine model. We here have demonstrated the ability of Haralick textural features to quantitatively characterize the evolution of textural components (e.g. contrast, homogeneity, entropy) within Raman maps of radiation-induced glycogen production in murine tumours. Our results indicate that Haralick feature calculations provide a new, quantitative assessment within a radiobiological context and may, in future studies, help quantify the extent of radiation response. In turn, such quantification may be valuable in the long term goal of personalized radiation therapy response monitoring.

## Supporting information

S1 Fig Top panel: PC component 1 from the Raman data analysis of irradiated murine tumours. Bottom panel: Raman spectrum of pure glycogen.
(TIFF)

S2 Fig Raman maps with pixel intensities equal to the scaled and discretized PC1 scores (glycogen production) for control unirradiated tumour sections. (TIFF)

S3 Fig Raman maps with pixel intensities equal to the scaled and discretized PC1 scores (glycogen production) for tumour sections irradiated to 5Gy. (TIFF)

S4 Fig Raman maps with pixel intensities equal to the scaled and discretized PC1 scores (glycogen production) for tumour sections irradiated to 15Gy. (TIFF)
