# Supplementary figures and images for: Haralick texture feature analysis for quantifying radiation response heterogeneity in murine models observed using Raman spectroscopic mapping
Source: PLoS One. 2019 Feb 15;14(2):e0212225. doi: 10.1371/journal.pone.0212225 (PMC6377107; doi:10.1371/journal.pone.0212225)

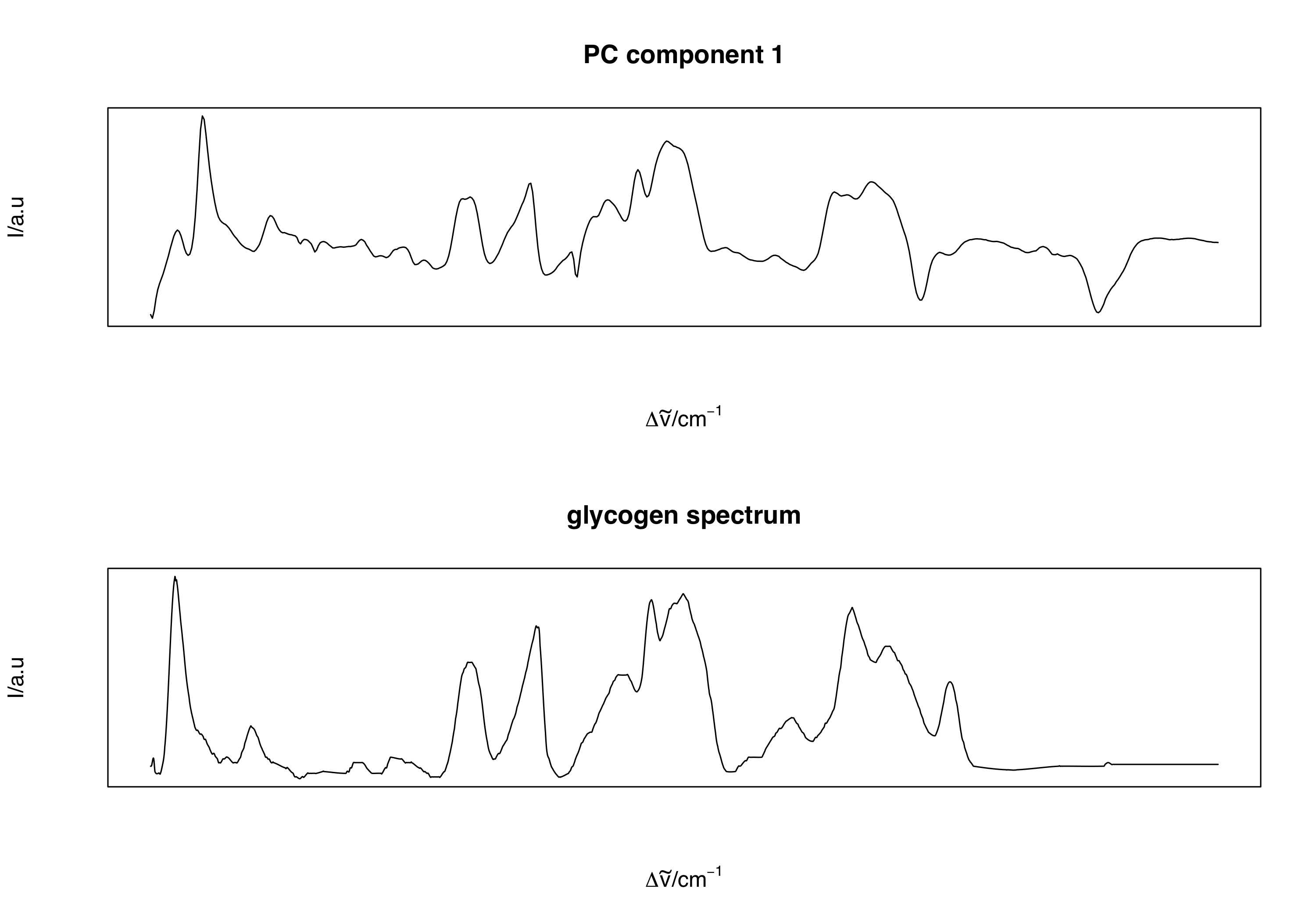

Supplement: S1 Fig — Top panel: PC component 1 from the Raman data analysis of irradiated murine tumours. Bottom panel: Raman spectrum of pure glycogen. (TIFF) [file pone.0212225.s001.tiff]

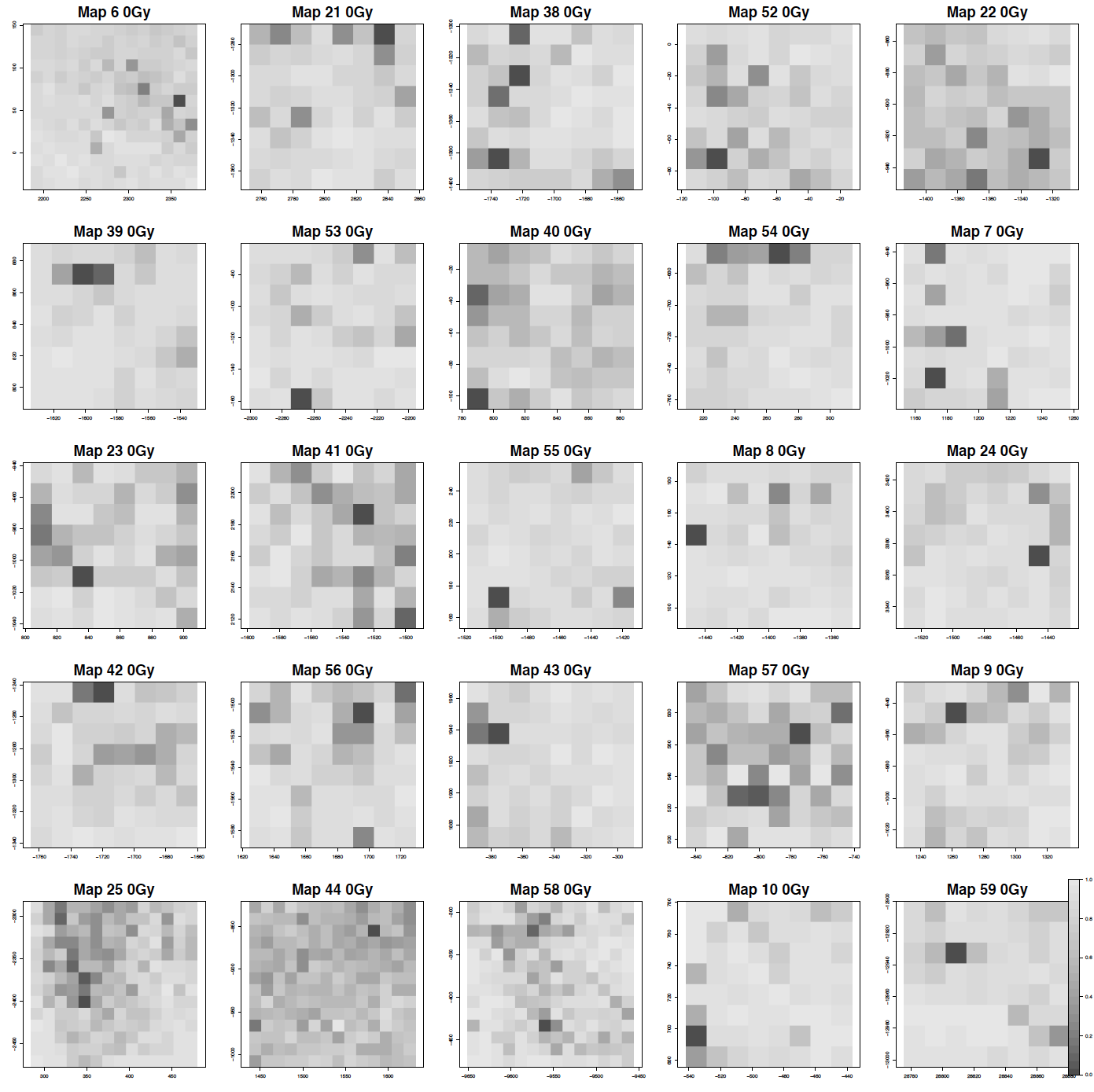

Supplement: S2 Fig — (TIFF) [file pone.0212225.s002.tiff]

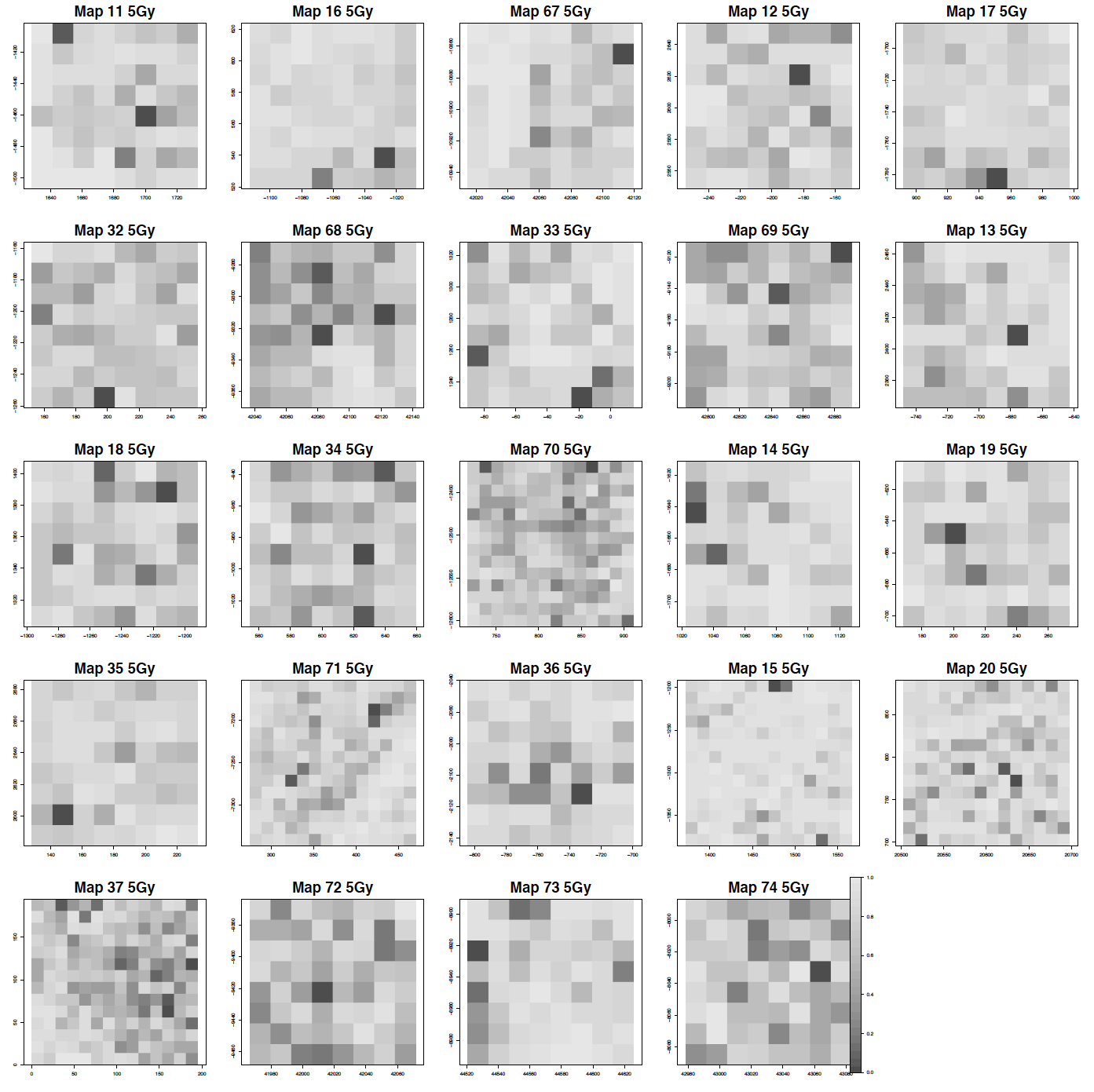

Supplement: S3 Fig — (TIFF) [file pone.0212225.s003.tiff]

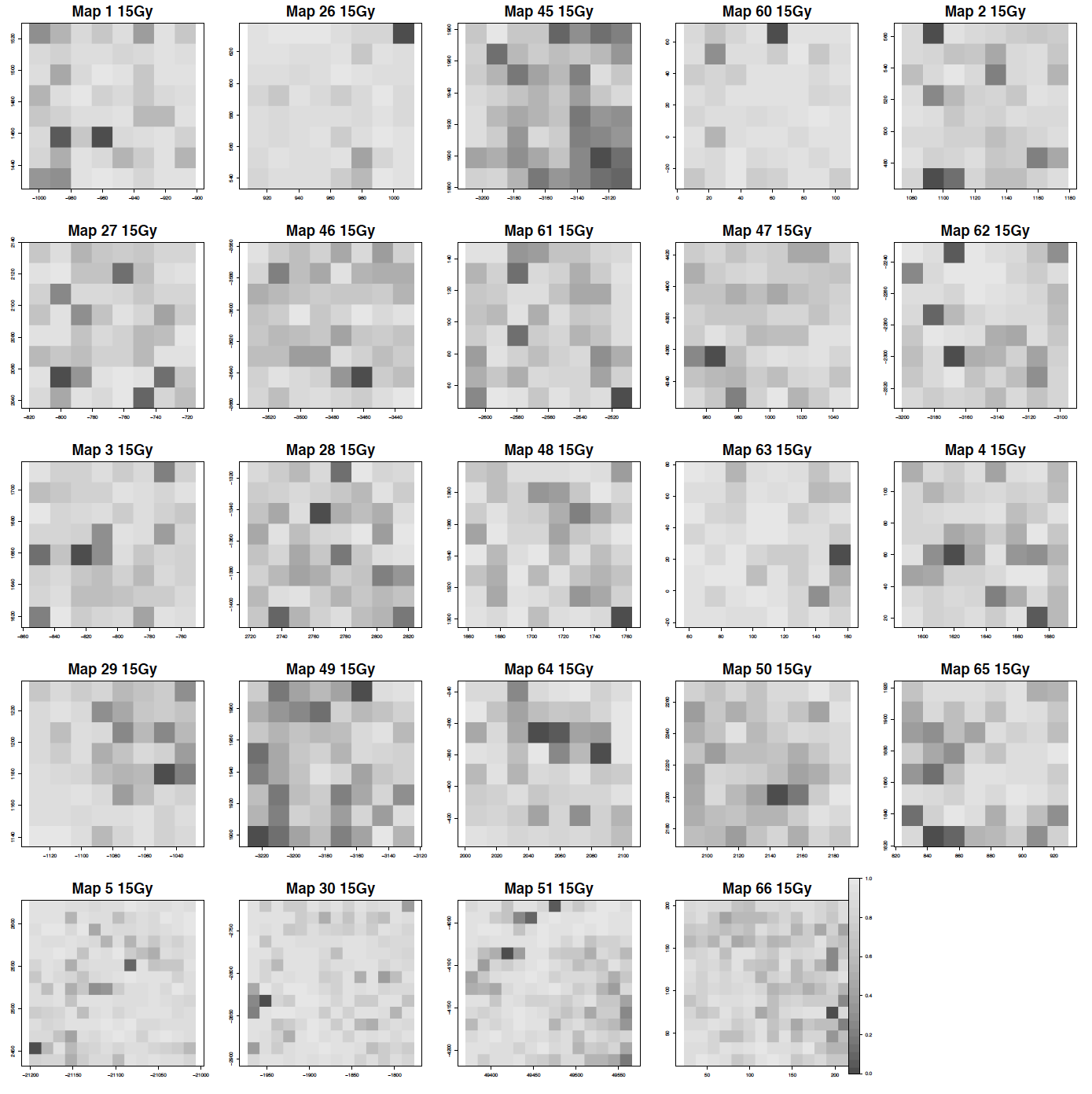

Supplement: S4 Fig — (TIFF) [file pone.0212225.s004.tiff]
